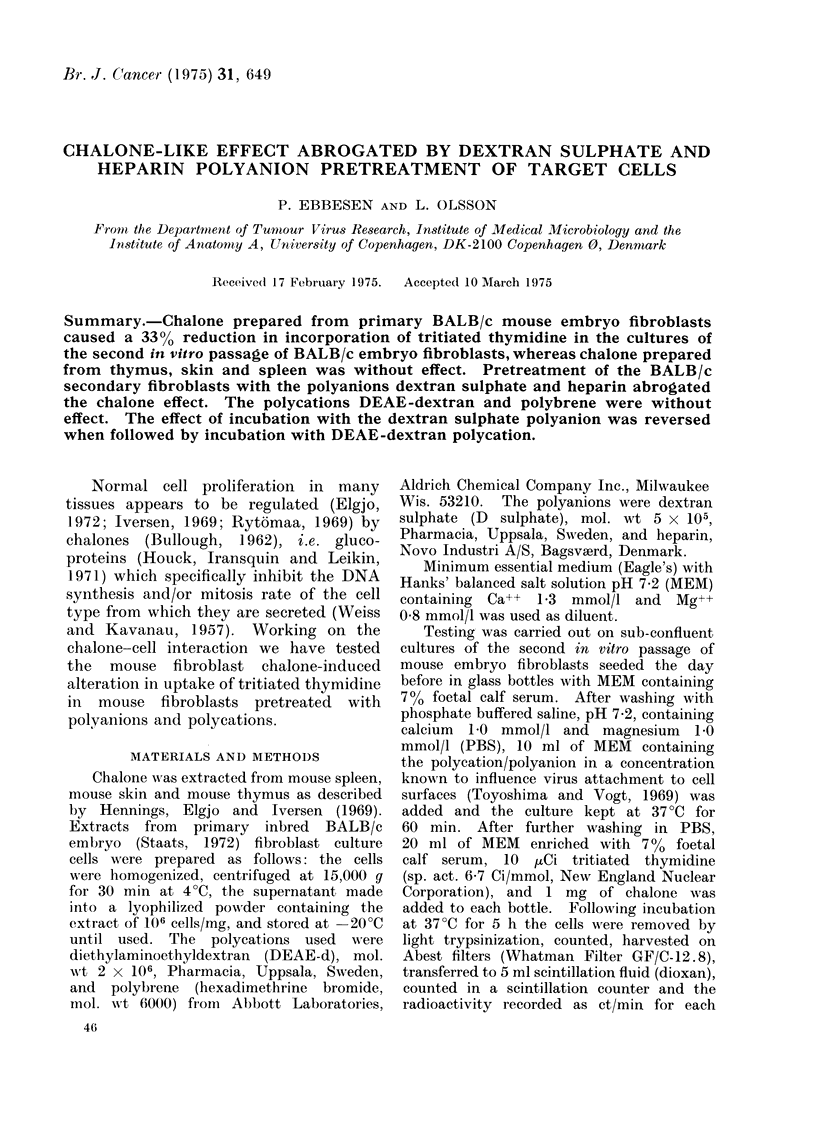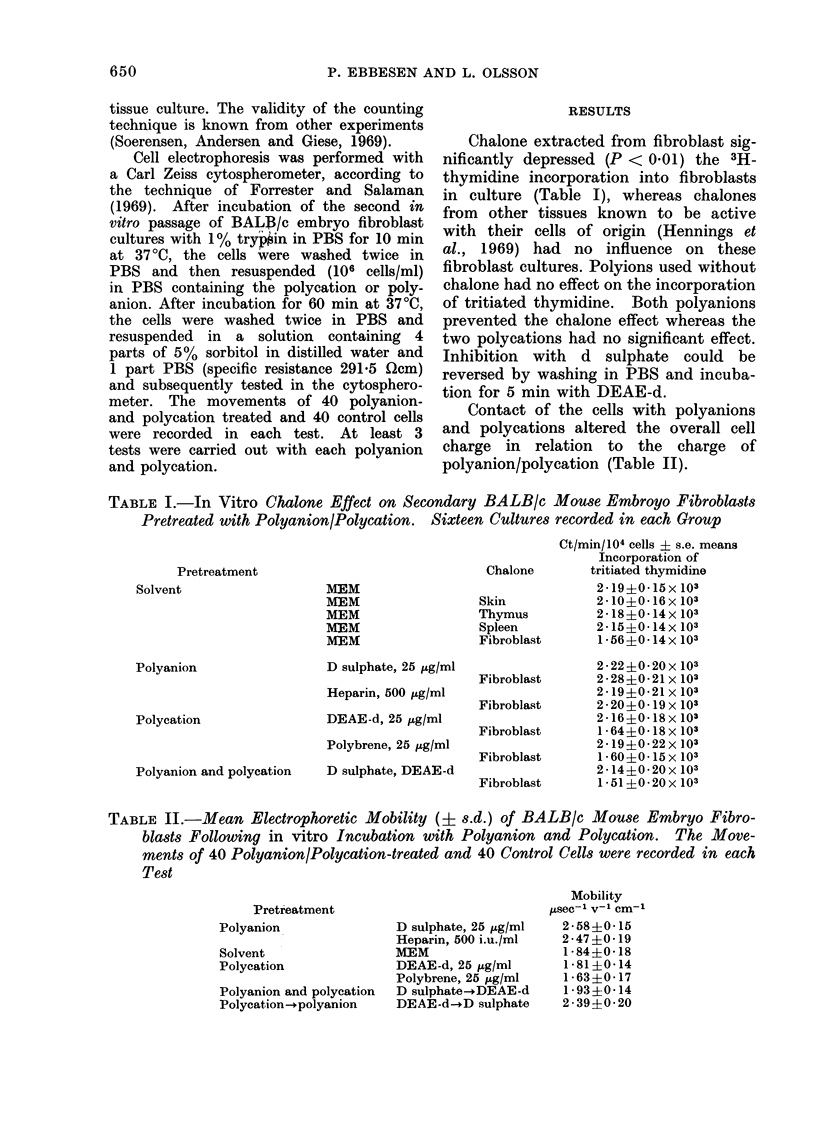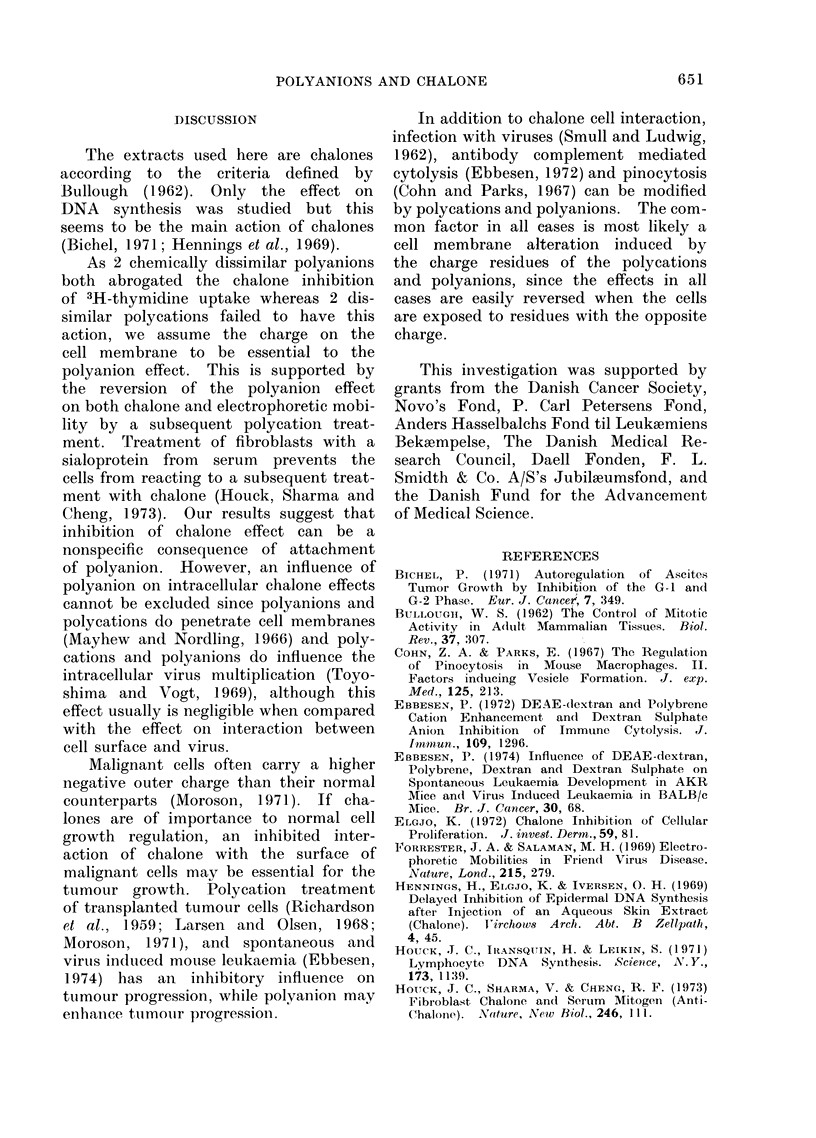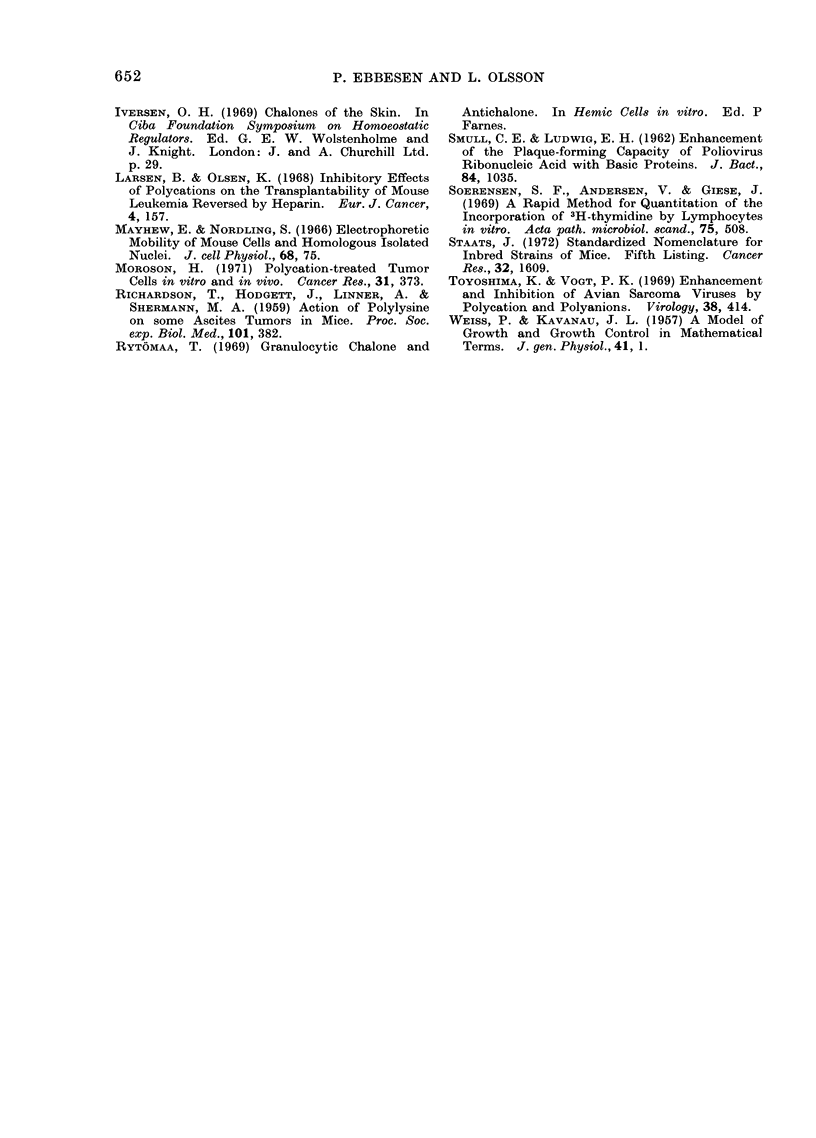# Chalone-like effect abrogated by dextran sulphate and heparin polyanion pretreatment of target cells.

**DOI:** 10.1038/bjc.1975.111

**Published:** 1975-06

**Authors:** P. Ebbesen, L. Olsson

## Abstract

Chalone prepared from primary BALB/c mouse embryo fibroblasts caused a 33% reduction in incorporation of tritiated thymidine in the cultures of the second in vitro passage of BALB/c embryo fibroblasts, whereas chalone prepared from thymus, skin and spleen was without effect. Pretreatment of BALB/c secondary fibroblasts with the polyanions dextran sulphate and heparin abrogated the chalone effect. The polycations DEAE-dextran and polybrene were without effect. The effect of incubation with the dextran sulphate polyanion was reversed when followed by incubation with DEAE-dextran polycation.


*Br. J. Cancer* (1975) **31**, 649

### CHALONE-LIKE EFFECT ABROGATED BY DEXTRAN SULPHATE AND HEPARIN POLYANION PRETREATMENT OF TARGET CELLS

P. EBBESEN AND L. OLSSON

*From the Department of Tumour Virus Research, Institute of Medical Microbiology and the Institute of Anatomy A, University of Copenhagen, DK-2100 Copenhagen Ø, Denmark*

Received 17 February 1975. Accepted 10 March 1975

**Summary.—Chalone prepared from primary BALB/c mouse embryo fibroblasts caused a 33% reduction in incorporation of tritiated thymidine in the cultures of the second *in vitro* passage of BALB/c embryo fibroblasts, whereas chalone prepared from thymus, skin and spleen was without effect. Pretreatment of the BALB/c secondary fibroblasts with the polyanions dextran sulphate and heparin abrogated the chalone effect. The polycations DEAE-dextran and polybrene were without effect. The effect of incubation with the dextran sulphate polyanion was reversed when followed by incubation with DEAE-dextran polycation.**

Normal cell proliferation in many tissues appears to be regulated (Elgjo, 1972; Iversen, 1969; Rytömaa, 1969) by chalones (Bullough, 1962), *i.e.* glucoproteins (Houck, Iransquin and Leikin, 1971) which specifically inhibit the DNA synthesis and/or mitosis rate of the cell type from which they are secreted (Weiss and Kavanau, 1957). Working on the chalone–cell interaction we have tested the mouse fibroblast chalone-induced alteration in uptake of tritiated thymidine in mouse fibroblasts pretreated with polyanions and polycations.

### MATERIALS AND METHODS

Chalone was extracted from mouse spleen, mouse skin and mouse thymus as described by Hennings, Elgjo and Iversen (1969). Extracts from primary inbred BALB/c embryo (Staats, 1972) fibroblast culture cells were prepared as follows: the cells were homogenized, centrifuged at 15,000 *g* for 30 min at 4°C, the supernatant made into a lyophilized powder containing the extract of $10^6$ cells/mg, and stored at −20°C until used. The polycations used were diethylaminoethyldextran (DEAE-d), mol. wt $2 \times 10^6$, Pharmacia, Uppsala, Sweden, and polybrene (hexadimethrine bromide, mol. wt 6000) from Abbott Laboratories, Aldrich Chemical Company Inc., Milwaukee Wis. 53210. The polyanions were dextran sulphate (D sulphate), mol. wt $5 \times 10^5$, Pharmacia, Uppsala, Sweden, and heparin, Novo Industri A/S, Bagsværd, Denmark.

Minimum essential medium (Eagle's) with Hanks' balanced salt solution pH 7·2 (MEM) containing $Ca^{++}$ 1·3 mmol/l and $Mg^{++}$ 0·8 mmol/l was used as diluent.

Testing was carried out on sub-confluent cultures of the second *in vitro* passage of mouse embryo fibroblasts seeded the day before in glass bottles with MEM containing 7% foetal calf serum. After washing with phosphate buffered saline, pH 7·2, containing calcium 1·0 mmol/l and magnesium 1·0 mmol/l (PBS), 10 ml of MEM containing the polycation/polyanion in a concentration known to influence virus attachment to cell surfaces (Toyoshima and Vogt, 1969) was added and the culture kept at 37°C for 60 min. After further washing in PBS, 20 ml of MEM enriched with 7% foetal calf serum, 10 μCi tritiated thymidine (sp. act. 6·7 Ci/mmol, New England Nuclear Corporation), and 1 mg of chalone was added to each bottle. Following incubation at 37°C for 5 h the cells were removed by light trypsinization, counted, harvested on Abest filters (Whatman Filter GF/C-12.8), transferred to 5 ml scintillation fluid (dioxan), counted in a scintillation counter and the radioactivity recorded as ct/min for each tissue culture. The validity of the counting technique is known from other experiments (Soerensen, Andersen and Giese, 1969).

Cell electrophoresis was performed with a Carl Zeiss cytospherometer, according to the technique of Forrester and Salaman (1969). After incubation of the second *in vitro* passage of BALB/c embryo fibroblast cultures with 1% trypsin in PBS for 10 min at 37°C, the cells were washed twice in PBS and then resuspended ($10^6$ cells/ml) in PBS containing the polycation or polyanion. After incubation for 60 min at 37°C, the cells were washed twice in PBS and resuspended in a solution containing 4 parts of 5% sorbitol in distilled water and 1 part PBS (specific resistance 291·5 Ωcm) and subsequently tested in the cytospherometer. The movements of 40 polyanion- and polycation treated and 40 control cells were recorded in each test. At least 3 tests were carried out with each polyanion and polycation.

### RESULTS

Chalone extracted from fibroblast significantly depressed ($P < 0{\cdot}01$) the $^3$H-thymidine incorporation into fibroblasts in culture (Table I), whereas chalones from other tissues known to be active with their cells of origin (Hennings *et al.*, 1969) had no influence on these fibroblast cultures. Polyions used without chalone had no effect on the incorporation of tritiated thymidine. Both polyanions prevented the chalone effect whereas the two polycations had no significant effect. Inhibition with d sulphate could be reversed by washing in PBS and incubation for 5 min with DEAE-d.

Contact of the cells with polyanions and polycations altered the overall cell charge in relation to the charge of polyanion/polycation (Table II).

TABLE I.—In Vitro *Chalone Effect on Secondary BALB/c Mouse Embroyo Fibroblasts Pretreated with Polyanion/Polycation. Sixteen Cultures recorded in each Group*

| Pretreatment | | Chalone | Ct/min/$10^4$ cells ± s.e. means Incorporation of tritiated thymidine |
|---|---|---|---|
| Solvent | MEM | | $2{\cdot}19 \pm 0{\cdot}15 \times 10^3$ |
| | MEM | Skin | $2{\cdot}10 \pm 0{\cdot}16 \times 10^3$ |
| | MEM | Thymus | $2{\cdot}18 \pm 0{\cdot}14 \times 10^3$ |
| | MEM | Spleen | $2{\cdot}15 \pm 0{\cdot}14 \times 10^3$ |
| | MEM | Fibroblast | $1{\cdot}56 \pm 0{\cdot}14 \times 10^3$ |
| Polyanion | D sulphate, 25 μg/ml | | $2{\cdot}22 \pm 0{\cdot}20 \times 10^3$ |
| | | Fibroblast | $2{\cdot}28 \pm 0{\cdot}21 \times 10^3$ |
| | Heparin, 500 μg/ml | | $2{\cdot}19 \pm 0{\cdot}21 \times 10^3$ |
| | | Fibroblast | $2{\cdot}20 \pm 0{\cdot}19 \times 10^3$ |
| Polycation | DEAE-d, 25 μg/ml | | $2{\cdot}16 \pm 0{\cdot}18 \times 10^3$ |
| | | Fibroblast | $1{\cdot}64 \pm 0{\cdot}18 \times 10^3$ |
| | Polybrene, 25 μg/ml | | $2{\cdot}19 \pm 0{\cdot}22 \times 10^3$ |
| | | Fibroblast | $1{\cdot}60 \pm 0{\cdot}15 \times 10^3$ |
| Polyanion and polycation | D sulphate, DEAE-d | | $2{\cdot}14 \pm 0{\cdot}20 \times 10^3$ |
| | | Fibroblast | $1{\cdot}51 \pm 0{\cdot}20 \times 10^3$ |

TABLE II.—*Mean Electrophoretic Mobility (± s.d.) of BALB/c Mouse Embryo Fibroblasts Following* in vitro *Incubation with Polyanion and Polycation. The Movements of* 40 *Polyanion/Polycation-treated and* 40 *Control Cells were recorded in each Test*

| Pretreatment | | Mobility μsec$^{-1}$ v$^{-1}$ cm$^{-1}$ |
|---|---|---|
| Polyanion | D sulphate, 25 μg/ml | $2{\cdot}58 \pm 0{\cdot}15$ |
| | Heparin, 500 i.u./ml | $2{\cdot}47 \pm 0{\cdot}19$ |
| Solvent | MEM | $1{\cdot}84 \pm 0{\cdot}18$ |
| Polycation | DEAE-d, 25 μg/ml | $1{\cdot}81 \pm 0{\cdot}14$ |
| | Polybrene, 25 μg/ml | $1{\cdot}63 \pm 0{\cdot}17$ |
| Polyanion and polycation | D sulphate→DEAE-d | $1{\cdot}93 \pm 0{\cdot}14$ |
| Polycation→polyanion | DEAE-d→D sulphate | $2{\cdot}39 \pm 0{\cdot}20$ |

### DISCUSSION

The extracts used here are chalones according to the criteria defined by Bullough (1962). Only the effect on DNA synthesis was studied but this seems to be the main action of chalones (Bichel, 1971; Hennings *et al.*, 1969).

As 2 chemically dissimilar polyanions both abrogated the chalone inhibition of $^{3}$H-thymidine uptake whereas 2 dissimilar polycations failed to have this action, we assume the charge on the cell membrane to be essential to the polyanion effect. This is supported by the reversion of the polyanion effect on both chalone and electrophoretic mobility by a subsequent polycation treatment. Treatment of fibroblasts with a sialoprotein from serum prevents the cells from reacting to a subsequent treatment with chalone (Houck, Sharma and Cheng, 1973). Our results suggest that inhibition of chalone effect can be a nonspecific consequence of attachment of polyanion. However, an influence of polyanion on intracellular chalone effects cannot be excluded since polyanions and polycations do penetrate cell membranes (Mayhew and Nordling, 1966) and polycations and polyanions do influence the intracellular virus multiplication (Toyoshima and Vogt, 1969), although this effect usually is negligible when compared with the effect on interaction between cell surface and virus.

Malignant cells often carry a higher negative outer charge than their normal counterparts (Moroson, 1971). If chalones are of importance to normal cell growth regulation, an inhibited interaction of chalone with the surface of malignant cells may be essential for the tumour growth. Polycation treatment of transplanted tumour cells (Richardson *et al.*, 1959; Larsen and Olsen, 1968; Moroson, 1971), and spontaneous and virus induced mouse leukaemia (Ebbesen, 1974) has an inhibitory influence on tumour progression, while polyanion may enhance tumour progression.

In addition to chalone cell interaction, infection with viruses (Smull and Ludwig, 1962), antibody complement mediated cytolysis (Ebbesen, 1972) and pinocytosis (Cohn and Parks, 1967) can be modified by polycations and polyanions. The common factor in all cases is most likely a cell membrane alteration induced by the charge residues of the polycations and polyanions, since the effects in all cases are easily reversed when the cells are exposed to residues with the opposite charge.

This investigation was supported by grants from the Danish Cancer Society, Novo's Fond, P. Carl Petersens Fond, Anders Hasselbalchs Fond til Leukæmiens Bekæmpelse, The Danish Medical Research Council, Daell Fonden, F. L. Smidth & Co. A/S's Jubilæumsfond, and the Danish Fund for the Advancement of Medical Science.

### REFERENCES

BICHEL, P. (1971) Autoregulation of Ascites Tumor Growth by Inhibition of the G-1 and G-2 Phase. *Eur. J. Cancer*, **7**, 349.

BULLOUGH, W. S. (1962) The Control of Mitotic Activity in Adult Mammalian Tissues. *Biol. Rev.*, **37**, 307.

COHN, Z. A. & PARKS, E. (1967) The Regulation of Pinocytosis in Mouse Macrophages. II. Factors inducing Vesicle Formation. *J. exp. Med.*, **125**, 213.

EBBESEN, P. (1972) DEAE-dextran and Polybrene Cation Enhancement and Dextran Sulphate Anion Inhibition of Immune Cytolysis. *J. Immun.*, **109**, 1296.

EBBESEN, P. (1974) Influence of DEAE-dextran, Polybrene, Dextran and Dextran Sulphate on Spontaneous Leukaemia Development in AKR Mice and Virus Induced Leukaemia in BALB/c Mice. *Br. J. Cancer*, **30**, 68.

ELGJO, K. (1972) Chalone Inhibition of Cellular Proliferation. *J. invest. Derm.*, **59**, 81.

FORRESTER, J. A. & SALAMAN, M. H. (1969) Electrophoretic Mobilities in Friend Virus Disease. *Nature, Lond.*, **215**, 279.

HENNINGS, H., ELGJO, K. & IVERSEN, O. H. (1969) Delayed Inhibition of Epidermal DNA Synthesis after Injection of an Aqueous Skin Extract (Chalone). *Virchows Arch. Abt. B Zellpath*, **4**, 45.

HOUCK, J. C., IRANSQUIN, H. & LEIKIN, S. (1971) Lymphocyte DNA Synthesis. *Science, N.Y.*, **173**, 1139.

HOUCK, J. C., SHARMA, V. & CHENG, R. F. (1973) Fibroblast Chalone and Serum Mitogen (Anti-Chalone). *Nature, New Biol.*, **246**, 111.

IVERSEN, O. H. (1969) Chalones of the Skin. In *Ciba Foundation Symposium on Homoeostatic Regulators*. Ed. G. E. W. Wolstenholme and J. Knight. London: J. and A. Churchill Ltd. p. 29.

LARSEN, B. & OLSEN, K. (1968) Inhibitory Effects of Polycations on the Transplantability of Mouse Leukemia Reversed by Heparin. *Eur. J. Cancer*, **4**, 157.

MAYHEW, E. & NORDLING, S. (1966) Electrophoretic Mobility of Mouse Cells and Homologous Isolated Nuclei. *J. cell Physiol.*, **68**, 75.

MOROSON, H. (1971) Polycation-treated Tumor Cells *in vitro* and *in vivo*. *Cancer Res.*, **31**, 373.

RICHARDSON, T., HODGETT, J., LINNER, A. & SHERMANN, M. A. (1959) Action of Polylysine on some Ascites Tumors in Mice. *Proc. Soc. exp. Biol. Med.*, **101**, 382.

RYTÖMAA, T. (1969) Granulocytic Chalone and Antichalone. In *Hemic Cells in vitro*. Ed. P Farnes.

SMULL, C. E. & LUDWIG, E. H. (1962) Enhancement of the Plaque-forming Capacity of Poliovirus Ribonucleic Acid with Basic Proteins. *J. Bact.*, **84**, 1035.

SOERENSEN, S. F., ANDERSEN, V. & GIESE, J. (1969) A Rapid Method for Quantitation of the Incorporation of $^3$H-thymidine by Lymphocytes *in vitro*. *Acta path. microbiol. scand.*, **75**, 508.

STAATS, J. (1972) Standardized Nomenclature for Inbred Strains of Mice. Fifth Listing. *Cancer Res.*, **32**, 1609.

TOYOSHIMA, K. & VOGT, P. K. (1969) Enhancement and Inhibition of Avian Sarcoma Viruses by Polycation and Polyanions. *Virology*, **38**, 414.

WEISS, P. & KAVANAU, J. L. (1957) A Model of Growth and Growth Control in Mathematical Terms. *J. gen. Physiol.*, **41**, 1.